# Supplementary material for: Mapping the evolution of fertility support policies in China: A content and instrumental analysis
Source: PLoS One. 2025 Oct 9;20(10):e0332137. doi: 10.1371/journal.pone.0332137 (PMC12510515; doi:10.1371/journal.pone.0332137)
Supplement: S1 Appendix — (ZIP) [file pone.0332137.s001.zip › S1 Appendix. 226 original policy documents/213-第十二届全国人民代表大会第五次会议关于2016年国民经济和社会发展计划执行情况与2017年国民经济和社会发展计划的决议(FBM-CLI-1-291460).doc]

第十二届全国人民代表大会第五次会议关于2016年国民经济和社会发展计划执行情况与2017年国民经济和社会发展计划的决议

**发布部门：** [全国人民代表大会](https://www.pkulaw.com/law/chl?Aggs.IssueDepartment=101)

**发布日期：**2017.03.15

**实施日期：**2017.03.15

**时效性：** [现行有效](https://www.pkulaw.com/law/chl?Aggs.TimelinessDic=01)

**效力级别：** [工作文件](https://www.pkulaw.com/law/chl?Aggs.EffectivenessDic=XA0105)

**法规类别：** [人大议事](https://www.pkulaw.com/law/chl?Aggs.Category=00106) [营商环境优化](https://dobusiness.pkulaw.com/)

**专题分类：** [长江经济带发展](https://www.pkulaw.com/law/chl?Aggs.SpecialType=022)

第十二届全国人民代表大会第五次会议关于2016年国民经济和社会发展计划执行情况与2017年国民经济和社会发展计划的决议
（2017年3月15日第十二届全国人民代表大会第五次会议通过）

　　第十二届全国人民代表大会第五次会议审查了国务院提出的《关于2016年国民经济和社会发展计划执行情况与2017年国民经济和社会发展计划草案的报告》及2017年国民经济和社会发展计划草案，同意全国人民代表大会财政经济委员会的审查结果报告。会议决定，批准《关于2016年国民经济和社会发展计划执行情况与2017年国民经济和社会发展计划草案的报告》，批准2017年国民经济和社会发展计划。

关于2016年国民经济和社会发展计划
执行情况与2017年国民经济和
社会发展计划草案的报告
--2017年3月5日在第十二届全国人民代表大会第五次会议上
国家发展和改革委员会

各位代表：
　　受国务院委托，现将2016年国民经济和社会发展计划执行情况与2017年国民经济和社会发展计划草案提请十二届全国人大五次会议审议，并请全国政协各位委员提出意见。
　　**一、**2016年国民经济和社会发展计划执行情况
　　去年以来，国内外环境复杂严峻，世界经济艰难复苏，国内经济下行压力依然较大。在以习近平同志为核心的党中央坚强领导下，各地区各部门坚持稳中求进工作总基调，坚持新发展理念，认真执行十二届全国人大四次会议审议批准的2016年国民经济和社会发展计划，落实全国人大财政经济委员会的审查意见，以推进供给侧结构性改革为主线，适度扩大总需求，坚定推进改革，妥善应对风险挑战，引导形成良好社会预期，扎实做好各项工作，经济社会保持平稳健康发展，“十三五”实现良好开局，计划执行情况总体是好的。
　　（一）创新和加强宏观调控，经济运行保持在合理区间。在区间调控基础上，加强定向调控、相机调控，积极的财政政策力度加大，稳健的货币政策灵活适度，确保经济平稳运行。国内生产总值达到74.41万亿元，增长6.7%，符合预期。

　　一是消费基础作用进一步增强。促进消费带动转型升级的行动方案出台，“十大扩消费行动”全面推进。促进绿色消费、实体零售创新转型、交通物流融合发展的政策出台实施。[进一步扩大旅游文化体育健康养老教育培训等领域消费的意见](https://www.pkulaw.com/chl/23dc0796e7b95b5fbdfb.html)发布实施，服务消费蓬勃兴起，汽车等实物消费扩大升级。[激发重点群体活力带动城乡居民增收的实施意见](https://www.pkulaw.com/chl/4495c185fd2553d5bdfb.html)出台，居民消费能力持续提升。社会消费品零售总额增长10.4%。消费在经济增长中发挥主要拉动作用，贡献率达到64.6%，消费和投资的比例关系进一步改善。

　　二是投资保持稳定增长。围绕补短板、调结构、增供给，努力扩大合理有效投资。中央预算内投资结构继续优化，政府投资项目储备库和三年滚动投资计划初步形成，重大工程建设加快推进。着力调动民间投资积极性，制定促进民间投资“26条”政策，加大政府和社会资本合作（PPP）模式推广力度。全社会固定资产投资增长7.9%，民间投资占固定资产投资（不含农户）比重为61.2%。

　　三是就业形势总体较好。基层就业社保服务设施和公共实训基地建设加快推进，高校毕业生和化解过剩产能职工就业服务工作力度加大，支持农民工等人员返乡创业试点持续推进。全年城镇新增就业1314万人，年末城镇登记失业率4.02%。

　　四是价格总水平基本稳定。大宗商品价格调控进一步强化，生猪价格调控有效开展，汛期、重点节假日蔬菜等重要商品价格监测预警和调控监管加强。价格监管和反垄断执法强力推进，先后查处了数起反垄断案件。全年居民消费价格上涨2.0%。
　　五是各类风险挑战得到妥善应对。加强对外投资活动真实性审查，引导对外投资健康有序发展。按照市场化、法治化原则防范化解债券违约风险。因城因地施策，分类调控房地产市场。防范应对部分地区特别是长江流域发生的严重洪涝等灾害，及时有力开展抢险救灾，最大限度降低了灾害损失，恢复重建有序进行。
　　（二）扎实推进供给侧结构性改革，“三去一降一补”五大重点任务初见成效。加强政策引导和支持，建立工作推进机制，去产能、去库存、去杠杆、降成本、补短板取得初步成效。
　　一是去产能年度任务提前超额完成。《[国务院关于钢铁行业化解过剩产能实现脱困发展的意见](https://www.pkulaw.com/chl/6b30c15e5b309e95bdfb.html)》（国发〔2016〕6号）和《[国务院关于煤炭行业化解过剩产能实现脱困发展的意见](https://www.pkulaw.com/chl/757711137ddfadbcbdfb.html)》（国发〔2016〕7号）印发实施。组织开展了淘汰落后、违法违规建设项目清理、联合执法三个专项行动，严格控制新增产能，加快淘汰落后产能，有序引导过剩产能退出。积极做好职工安置和债务处置，推进企业兼并重组、转型升级和布局优化。及时妥善应对供需调整、价格波动影响。2016年退出钢铁和煤炭产能分别超过6500万吨和2.9亿吨，超额完成年度目标任务。钢铁、煤炭企业效益转好，拖欠减少，现金流紧张、安全投入欠账、工资欠发缓发等问题得到一定缓解，行业运行状况和市场预期有所改善。
　　二是去库存加快推进。推动农业转移人口落户城镇，满足新市民住房需求，年末商品住宅待售面积比上年末减少4991万平方米。棚户区住房改造货币化安置比例进一步提高，全年货币化安置294万户，占全年棚改开工量的48.5%，比2015年提高18.6个百分点。
　　三是去杠杆成效初步显现。 《[国务院关于积极稳妥降低企业杠杆率的意见](https://www.pkulaw.com/chl/06a05e79c95e8bc0bdfb.html)》（国发〔2016〕54号）印发实施。采取兼并重组、市场化法治化债转股、发展股权融资等综合性措施，积极稳妥降低企业杠杆率。启动市场化银行债权转股权，截至2016年底，多家商业银行通过实施机构共选择了资产负债率偏高但具有发展前景的行业龙头企业20家，自主协商达成债转股框架协议，协议金额超过2500亿元。2016年末，全国规模以上工业企业资产负债率55.8%，同比下降0.4个百分点。
　　四是降成本取得明显成效。《[国务院关于印发降低实体经济企业成本工作方案的通知](https://www.pkulaw.com/chl/d2556c4e3b1e4b94bdfb.html)》（国发〔2016〕48号）印发实施。持续推进简政放权放管结合优化服务改革，降低制度性交易成本。全面推开营改增试点，阶段性降低“五险一金”缴费比例。实施煤电价格联动，推进输配电价改革，扩大电力直接交易规模，完善基本电价执行方式，降低企业用能成本。清理规范进出口环节、金融等领域涉企收费，推进铁路货运体制改革，开展物流业降本增效专项行动，印发实施推进物流大通道建设行动计划。2016年，规模以上工业企业每百元主营业务收入中的成本同比降低0.1元，利润率同比提高0.19个百分点。
　　五是补短板力度加大。坚持既利当前又利长远，既重硬设施又重软能力，以市场化投融资方式带动银行贷款等资金投放，在脱贫攻坚、灾后水利恢复重建、社会事业、创新能力、新产业等关键领域和薄弱环节，抓好补短板建设。全年完成1000万以上农村贫困人口脱贫目标任务。

　　（三）改革开放取得新突破，经济社会发展活力进一步释放。一批具有标志性、关键性的重大改革方案出台实施，重要领域和关键环节改革取得突破性进展，开放型经济发展水平不断提升。
　　一是简政放权放管结合优化服务改革向纵深推进。在提前完成本届政府减少行政审批事项三分之一目标的基础上，去年又取消165项国务院部门及其指定地方实施的审批事项，清理规范192项审批中介服务事项、220项职业资格许可认定事项。第三次修订政府核准的投资项目目录。商事制度改革继续深化。全面推行“双随机、一公开”，增强事中事后监管的有效性，推进“互联网+政务服务”。市场准入负面清单制度改革试点开局良好。“放管服”四大平台建成运行。

　　二是投融资体制改革步伐加快。深化投融资体制改革意见、[企业投资项目核准和备案管理条例](https://www.pkulaw.com/chl/9545cd6cfc040dd1bdfb.html)出台，新一轮投融资体制改革全面展开。修订发布[中央预算内投资补助和贴息项目管理办法](https://www.pkulaw.com/chl/f0158d3f374b9244bdfb.html)，制定80个专项管理办法。济青、杭绍台等铁路吸引社会资本示范项目取得积极进展。
　　三是价格改革继续深化。输配电价改革试点实现所有省级电网全覆盖。建立天然气管道运输定价新机制，占消费总量80%以上的非居民用气门站价格主要由市场主导形成。石油天然气交易市场加快发展。90%左右的城市已推广居民阶梯水电气价。全面推进医疗服务价格改革。铁路、民航旅客票价市场化程度明显提高。农业水价综合改革稳步推进。完善稻谷和小麦最低收购价政策，棉花、大豆目标价格改革试点深入推进。

　　四是国有企业和重点行业改革稳步开展。国企改革“1+N”文件体系基本形成，加快剥离办社会职能和解决历史遗留问题工作方案等配套文件出台，深化国企改革九项重点任务、十项改革试点扎实推进。第一批混合所有制改革试点进入实施阶段。第一批国有资本投资公司试点取得阶段性进展。31个省（区、市）电改方案获批复，公布了首批105个增量配电业务改革试点项目。各省（区、市）国有林场改革实施方案和内蒙古、吉林、黑龙江重点国有林区改革实施方案均已制定完成，浙江、湖南、江西等6省国有林场改革试点顺利完成。盐业体制改革方案出台，全面放开食盐出厂、批发和零售价格。改革玉米收储制度，将东北地区玉米临时收储政策调整为市场化收购加补贴新机制。政策性粮食和储备棉库存消化进展顺利。
　　五是公平竞争市场环境加快形成。印发[完善产权保护制度依法保护产权的意见](https://www.pkulaw.com/chl/0d3cd162234f304dbdfb.html)，依法平等保护各种所有制经济权益。工业用地市场化配置改革试点稳步实施。[在市场体系建设中建立公平竞争审查制度的意见](https://www.pkulaw.com/chl/a87136f6ba497fd7bdfb.html)出台，从源头上预防政府部门制定出台限制竞争的政策措施。社会信用体系建设取得新进展，出台政务、个人、电子商务领域诚信建设指导意见，50多个部门在12个领域开展失信联合惩戒、3个领域开展守信联合激励。打击侵权假冒合力增强，查办违法犯罪案件17万余件。国内贸易流通体制改革发展综合试点稳步推进。
　　六是财税金融改革有序推进。 《[国务院关于推进中央与地方财政事权和支出责任划分改革的指导意见](https://www.pkulaw.com/chl/d7912543ce5b8429bdfb.html)》（国发〔2016〕49号）出台实施。全面推开营改增试点，将建筑业、房地产业、金融业、生活服务业纳入营改增范围，并将所有企业新增不动产所含增值税纳入抵扣范围。全面实施资源税从价计征改革，开展水资源税改革试点。深化国有商业银行和开发性、政策性金融机构改革。存款保险制度平稳运行。上海自贸试验区部分金融开放创新举措推广至广东、天津、福建自贸试验区。“深港通”开启。
　　七是社会领域改革加快推进。中央和国家机关公务用车制度改革全面完成，地方党政机关公车改革深入推进。行业协会商会与行政机关脱钩改革第二批试点启动实施。加快不动产统一登记制度落地，全国100%的地（市、州）、98%的县（市、区）实现“发新停旧”。国家科技计划管理改革继续深化，以增加知识价值为导向的分配政策出台，科技成果转化力度加大。教育领域综合改革向纵深推进。分级诊疗制度建设持续推进，城乡居民基本医疗保险制度整合取得实质性进展，个人卫生支出占卫生总费用的比重下降到28.9%。《[国务院办公厅关于全面放开养老服务市场提升养老服务质量的若干意见](https://www.pkulaw.com/chl/e639f039d2c852a4bdfb.html)》（国办发〔2016〕91号）印发实施。稳步推进机关事业单位养老保险制度改革。加快构建中国特色哲学社会科学，实施哲学社会科学创新工程。足球改革加快推进。

　　八是以“一带一路”建设为统领推动开放型经济水平不断提升。 “一带一路”建设进展快速。“六廊六路多国多港”主骨架建设稳步推进，战略对接、规划对接成效显著，中欧班列实现了统一品牌，累计开行近3000列。一批国际产能合作标志性工程落地，亚的斯亚贝巴-吉布提铁路正式通车，从投融资、技术标准到运营管理维护，全部采用中国标准，标志着中国铁路首次实现全产业链“走出去”。印尼雅万高铁、中老铁路、中泰铁路、马来西亚南部铁路、匈塞铁路、瓜达尔港等重大项目有序推进。外商投资便利化程度进一步提高，非金融类实际使用外资保持稳定。对外投资管理体制机制更加完善，非金融类对外投资继续增长。国际贸易“单一窗口”在沿海口岸全部启用，通关一体化、检验检疫一体化管理覆盖全国。全年货物贸易进出口总额下降0.9%，降幅比上年收窄6.1个百分点。服务贸易较快增长。人民币正式加入国际货币基金组织特别提款权（SDR）货币篮子。特别是成功主办二十国集团领导人杭州峰会，影响深远。

　　（四）深入实施创新驱动发展战略，经济发展新动能加快成长。 《[国家创新驱动发展战略纲要](https://www.pkulaw.com/chl/2483bb1a3139093dbdfb.html)》印发实施，取得一批重大科技成果，高技术产业、装备制造业、战略性新兴产业较快增长，创新对发展的支撑作用增强。
　　一是创新能力持续提升。由点及面、有序布局重大科技创新平台建设，深入推进8个区域全面创新改革试验。北京、上海建设科技创新中心迈出新步伐。河北·京南、浙江、宁波3个国家科技成果转移转化示范区启动建设。首个国家高速列车技术创新中心建成。完全自主知识产权中国标准高速列车正式投入运营。建成世界最大单口径射电望远镜（FAST）等一批重大科技基础设施，在量子通信、航空航天等方面取得一批重大科技成果。国家科技重大专项深入实施，科技创新2030-重大项目遴选确定。大力推动企业技术创新，落实好研发费用加计扣除、股权激励和技术入股所得税优惠、完善高新技术企业认定办法等政策。

　　二是大众创业万众创新广泛开展。28个国家双创示范基地建设全面推进。双创政策信息服务平台开通，双创白皮书发布。国家新兴产业创业投资引导基金、科技成果转化引导基金子基金和国家中小企业发展基金实体基金设立运行，投贷联动试点启动，创业担保贷款创新发展。第二届全国双创活动周及“创响中国”巡回接力活动成功举办。大型央企建设双创实体平台128个，印发实施进一步完善中央财政科研项目资金管理等政策的若干意见，科研院所和高校鼓励创新创业机制逐步完善，各类孵化器、专业化众创空间日趋成熟。全年平均每天新登记企业1.51万户。
　　三是新技术新产业新业态新模式蓬勃发展。工业机器人、集成电路、卫星应用、通用航空、生物等新产业快速发展。战略性新兴产业实现平稳增长，2016年，27个重点监测战略性新兴产业行业规模以上企业实现收入和利润分别增长11.32%和13.96%。“互联网+”行动和国家大数据战略深入推进，人工智能、虚拟现实、基因工程等新技术加速兴起，平台经济、分享经济、协同经济等新模式广泛渗透，线上线下融合、跨境电商、社交电商、智慧家庭、智能交通等新业态不断涌现。全年网上零售交易额近5.2万亿元，同比增长26.2%；其中，实物商品网上零售额占社会消费品零售总额的12.6%。
　　四是传统产业转型升级步伐加快。加快落实《中国制造2025》，组织实施增强制造业核心竞争力三年行动计划和制造业升级改造重大工程包，8个产业化实施方案顺利推进。铁路关键装备研发试验取得重大突破，226个智能制造综合标准化试验验证和新模式应用项目有序开展，国家机器人检测与评定中心建设进展顺利。先进制造产业投资基金设立。
　　五是服务业创新发展稳步推进。落实加快发展生产性服务业、生活性服务业的两个指导意见，服务业领域放宽市场准入实施规划出台，新一轮服务业综合改革试点启动。安排服务业发展引导资金，支持163个公共服务平台建设。第三产业增速继续超过第二产业，增加值占国内生产总值的比重提高到51.6%。
　　六是基础设施网络进一步完善。加快推进现代综合交通运输体系建设，交通基础设施网络总里程突破500万公里。交通提质增效、扶贫脱贫、重大基础设施建设“一二三百”工程出台实施，推进多式联运发展，促进通用航空业发展，推动交通物流融合发展。能源生产和消费革命战略出台，能源供应能力不断增强，非化石能源消费比重预计上升到13.3%，煤炭消费比重下降到62.0%。全国地级市基本建成光网城市，新一代信息基础设施更加完善，建成全球最大的4G网络。

　　（五）着力提升农业可持续发展能力，农业现代化建设取得新进展。农业农村发展继续保持稳中有进的良好态势，粮食总产量达到6.16亿吨，为经济社会发展大局提供了有力支撑。
　　一是农业生产能力继续提高。落实《全国新增1000亿斤粮食生产能力规划》，高标准农田建设加快推进，全面完成永久基本农田划定，国家粮食安全和重要农产品供给保障能力进一步增强。新建粮食仓容195亿斤。农业生产稳中调优，籽粒玉米种植面积调减2039万亩，粮改饲、粮改豆试点范围扩大，畜产品和水产品综合生产能力继续增强。农业绿色发展稳健起步，农业环境突出问题治理专项启动实施，农业可持续发展试验示范区启动创建，“一控两减三基本”行动取得阶段性成效，对农业面源污染、东北黑土地、农牧交错带已垦草原综合治理试点的支持力度加大。
　　二是农业农村基础设施不断完善。中小河流治理、小型病险水库除险加固、灌排骨干工程建设与配套改造和小型农田水利建设等进展顺利，新增高效节水灌溉面积2000万亩以上，农村饮水安全巩固提升工程启动实施。新一轮农村电网改造升级工程，包括小城镇中心村农网改造升级和平原地区农村机井通电等重点工程全面实施。农村交通基础设施服务水平不断提升，新改建农村公路29万公里。“宽带乡村”工程继续实施，电信普遍服务试点全面部署，农村信息基础设施进一步改善。
　　三是农村一二三产业融合发展进展顺利。农村产业融合发展“百县千乡万村”试点示范工程启动，137个试点示范县在优化发展布局、推进产城融合发展、构建现代农业产业体系、创新投融资机制等方面进行了积极探索。农村产业融合发展孵化园区加快建设，多元融合主体发展较快，打造了一批农业产业化龙头企业，农村新产业新业态蓬勃发展。
　　四是农村改革稳步推进。农村土地所有权、承包权、经营权分置办法出台实施，土地承包经营权确权登记颁证面积超过8亿亩。房地一体的农村宅基地、集体建设用地确权登记加快推进。农村集体产权制度改革意见出台，农村集体资产股份权能改革试点和土地承包权有偿退出试点积极开展。农村承包土地经营权和农民住房财产权抵押贷款试点稳妥有序推进。土地经营权流转和抵押规模不断扩大。各类新型经营主体发展壮大，数量超过270万家。
　　（六）深入实施三大战略和新型城镇化，城乡区域发展格局正在重塑优化。发挥三大战略、“四大板块”的叠加效应，促进城乡区域协调协同发展，提升新型城镇化质量和水平，新增长点增长极增长带加快发展壮大。
　　一是三大战略扎实有序推进。各省（区、市）主动融入丝绸之路经济带和21世纪海上丝绸之路建设，新疆、福建“一带一路”核心区建设成效初显。京津冀协同发展有序推进，北京城市副中心加快规划建设，一批疏解示范项目稳步实施，交通、生态、产业三个重点领域协同推进，一批重大改革举措落地。长江经济带发展规划纲要印发实施，坚持共抓大保护、不搞大开发，绿色生态廊道建设加快推进，覆盖上中下游地区的省际协商合作机制全面建立。

　　二是区域发展总体战略和差别化区域政策深入实施。加快新一轮西部大开发，一批重大标志性工程开工建设，中西部地区经济继续保持较快增长。出台实施新一轮东北振兴战略和若干重大政策举措，国企改革、投资营商环境改善等重点领域改革取得新进展。明确中部地区“一中心、四区”重要战略定位，长江中游城市群重要增长极作用强化。东部地区转型升级、开放创新、陆海统筹优势持续发挥，区域发展新动能新亮点不断涌现。出台促进区域协调发展的指导意见，差别化区域政策体系持续完善。国家综合配套改革试验区、国家级新区及各类开发区、承接产业转移示范区、产城融合示范区、临空经济示范区等重要功能平台建设有序推进。加快革命老区、民族地区、边疆地区和集中连片特困地区脱贫攻坚。完善支持政策措施，推进新疆、西藏、四省藏区经济社会发展和长治久安。

　　三是新型城镇化建设稳步推进。农业转移人口市民化步伐加快，户籍制度改革意见、居住证制度以及人地挂钩、支持农业转移人口市民化财政政策等关键性配套政策出台，全年进城落户人口达1600万人，常住人口城镇化率达到57.35%，户籍人口城镇化率达到41.2%。城市群规划建设有序推进，支持武汉、郑州建设国家中心城市。在全国开展新生中小城市和特色小（城）镇培育，国家新型城镇化综合试点范围扩大到2个省246个城市（镇）。61个中小城市综合改革试点取得积极成效。

　　（七）加强生态环境保护和能源资源节约，绿色发展初见成效。单位国内生产总值能耗和二氧化碳排放量分别下降5%和6.6%，超额完成全年目标，化学需氧量、氨氮、二氧化硫、氮氧化物排放量预计分别下降2.6%、2.9%、5.6%、4.0%，地级及以上城市空气质量优良天数比例同比提高2.1个百分点，74个重点城市细颗粒物（PM2.5）年均浓度下降9.1%，地表水达到或好于Ⅲ类水体比例同比提高1.8个百分点、劣Ⅴ类水体比例同比下降1.1个百分点，万元国内生产总值用水量下降5.6%。

　　一是生态文明建设取得新进展。[生态文明建设目标评价考核办法](https://www.pkulaw.com/chl/a845bded804ce877bdfb.html)印发实施。决定在福建省、江西省和贵州省开展国家生态文明试验区建设，生态文明先行示范区建设持续深化。健全国家自然资源资产管理体制试点方案、省以下环保机构监测监察执法垂直管理制度改革试点指导意见、控制污染物排放许可制实施方案、划定并严守生态保护红线若干意见、培育环境治理和生态保护市场主体意见等一批改革方案印发实施。在吉林等7省（市）开展生态环境损害赔偿制度改革试点。[全面推行河长制意见](https://www.pkulaw.com/chl/84c6622ed42e00f8bdfb.html)出台。生态保护补偿机制不断健全。新一轮退耕还林还草、重点防护林、石漠化综合治理、京津风沙源治理、水土流失综合治理等工程加快推进，湿地保护体系初步形成。开展中央环境保护督察。
　　二是主体功能区战略深入实施。国家重点生态功能区范围拓展到676个县及87个重点国有林区林业局，产业准入负面清单制度出台实施。省级海洋主体功能区规划编制实施工作在11个沿海省份展开。三江源、东北虎豹、大熊猫、神农架、武夷山、钱江源等国家公园体制试点方案印发实施。资源环境承载能力监测预警机制初步建立，先行在京津冀地区试点。市县“多规合一”试点深入推进，[省级空间规划试点方案](https://www.pkulaw.com/chl/40f2f91985be282fbdfb.html)印发实施。全国国土规划纲要、全国土地利用总体规划纲要调整方案印发实施。
　　三是节能减排持续推进。“十二五”省级人民政府节能目标责任评价考核结果公布，“十三五”能耗、水耗总量和强度“双控”制度建立实施，在浙江、福建、河南、四川4省开展用能权有偿使用和交易制度试点。循环经济发展加快，生产者责任延伸制度方案印发实施，产业园区循环化改造继续推进。印发《“十三五”节能环保产业发展规划》，构建绿色金融体系指导意见出台实施，发行各类绿色债券2296.6亿元，节能环保产业不断做大做强。
　　四是环境综合治理力度加大。《京津冀地区大气污染防治强化措施（2016-2017年）》发布实施，重点地区煤炭消费量进一步压减，加快燃煤电厂超低排放改造，全国累计淘汰黄标车和老旧车404.6万辆。制定实施2016-2017年长江经济带生态环境保护行动计划，完成11省（市）126个地级及以上城市全部319个集中式饮用水水源保护区划定，出台《全国重要饮用水水源地名录》，排查整治城市黑臭水体和非正规垃圾堆放点。深入开展农村生活垃圾治理专项行动，推进农村人居环境综合整治。土壤污染防治行动计划发布实施，出台污染地块土壤环境管理办法，启动14个土壤污染治理与修复试点项目和6个土壤污染综合防治先行区建设，加强重金属污染防控重点区域综合治理。全国矿山地质环境恢复和综合治理进一步加强。环境风险管控水平进一步提高，突发环境事件得到妥善处置。
　　五是应对气候变化工作不断强化。“十三五”控制温室气体排放工作方案印发实施。低碳省市、城（镇）、园区、社区等试点示范有序开展。碳排放权交易市场建设积极推进。城市适应气候变化行动方案发布实施。气候变化南南合作加快推进，“十百千”项目启动实施。首批签署和较早批准《巴黎协定》，创新性推动实现中美元首出席批准文书交存仪式，为推动《巴黎协定》尽早生效作出历史性贡献。建设性参加联合国气候变化马拉喀什会议并推动会议取得成功。
　　（八）加快发展社会事业和改善民生，人民生活水平持续提升。全力推进精准扶贫精准脱贫，加强社会政策托底，努力提高基本公共服务均等化水平。

　　一是脱贫攻坚战全面打响。《“十三五”脱贫攻坚规划》发布实施，全国财政专项扶贫资金投入超过1000亿元。印发实施“十三五”易地扶贫搬迁规划，22个省（区、市）全面启动易地扶贫搬迁项目，全年249万人易地扶贫搬迁建设任务如期完成。金融扶贫、特色产业扶贫、教育扶贫、交通扶贫、水利扶贫、旅游扶贫、光伏扶贫、电商扶贫、以工代赈等深入推进。东西部扶贫协作不断深化。推动集中连片特困地区“十三五”省级实施规划落地，对川陕等革命老区、赣南等原中央苏区振兴发展与脱贫攻坚支持力度持续加大。支持四川凉山、云南怒江、甘肃临夏等面临特殊困难的少数民族自治州加快建设小康社会进程。
　　二是社会保障能力不断加强。全国居民人均可支配收入实际增长6.3%，农村居民收入增幅连续7年高于城镇居民。退休人员基本养老金水平提高6.5%左右。基本医疗保险参保人数超过13亿，城乡居民基本医保补助标准由每人每年380元提高到420元。在全国范围内建立了困难残疾人生活补贴和重度残疾人护理补贴制度。
　　三是公共服务供给持续改善。学前教育毛入园率、九年义务教育巩固率、高中阶段毛入学率分别达到77.4%、93.4%和87.5%。高等教育毛入学率达到42.7%，超过中高收入国家平均水平。现代职业教育体系进一步完善和发展。城乡基本医疗服务体系进一步健全，基本公共卫生服务项目年人均财政补助达到45元，均等化水平明显提高。“健康中国2030”规划纲要发布实施，每千人口执业（助理）医师数增长到2.31人，每万人全科医生数增长到1.53人。全年出生人口达到1786万人，全面两孩政策平稳落地，生育服务保障进一步加强。公共文化服务体系建设继续加强，各省（区、市）均已出台本地区的基本公共文化服务实施标准。[实施中华优秀传统文化传承发展工程的意见](https://www.pkulaw.com/chl/0ce8bbbbea372123bdfb.html)等基础性制度出台。实施全民健身计划，体育健儿在里约奥运会、残奥会上再创佳绩。冰雪、水上、航空、山地户外等运动加快发展。

　　四是保障性安居工程建设扎实推进。加大中央预算内投资和中央财政专项资金支持力度，继续发挥企业债券直接融资作用，支持棚户区住房改造等保障性安居工程。2016年，完成棚户区住房改造600多万套，农村危房改造380多万户。
　　从计划指标运行情况看，经济增长、就业、价格总水平、国际收支平衡等主要指标保持在合理区间，科技创新、生态环保、民生保障等反映发展质量和效益的指标进一步改善，总的完成情况是好的。
　　19个约束性指标全部完成。43个预期性指标中，38个指标运行情况符合或高于预期，第一产业、全社会固定资产投资、货物进出口额、广义货币供应量（M2）增长率、城镇居民人均可支配收入5个指标运行值与预期值存在差距。需要说明的是，预期性指标的计划目标不是指令性的，也不是预测值，而是国家期望的发展目标，体现预期和政策导向，实际运行情况可能高于预期目标，也可能低于预期目标。部分指标运行值与预期目标存在差距，主要有以下原因：一是针对农产品阶段性供大于求、库存高企、价格低迷的形势，主动推进种养结构调整，部分主要农产品产量有所回落，因此第一产业增加值增幅略低于预期目标。二是受市场需求偏弱、投资回报下降、企业信心不强等因素综合影响，投资特别是民间投资、制造业投资下行压力较大，加上固定资产投资价格下跌持续时间和幅度超出预期，导致全社会固定资产投资增幅低于预期目标。三是受全球经济复苏乏力、国际贸易需求疲软、针对我国的保护主义加剧等影响，货物贸易进出口虽回稳向好，但仍难以实现预期目标。四是为供给侧结构性改革营造适宜的货币金融环境，在对实体经济提供足够支持的同时，货币供应切实体现了不搞“大水漫灌”和去杠杆、防风险、抑制资产价格泡沫的要求，因此广义货币供应量（M2）增速低于预期目标。五是从收入指标完成情况看，全国居民人均可支配收入增长与经济增长基本同步，受经济下行压力影响，部分行业从业人员工资性收入增幅放缓，导致城镇居民人均可支配收入增幅低于预期目标。
　　总之，在国内外形势错综复杂的情况下，我国经济增速继续走在世界前列，发展质量和效益提高，经济结构继续优化，人民生活水平持续改善，生态环境有所好转，成绩来之不易。这是党中央、国务院正确领导的结果，是各地区各部门共同努力的结果，是全国各族人民团结奋斗的结果。
　　同时，我们也清醒地认识到，世界经济仍然在深度调整，复苏动力不足，不稳定和不确定性将进一步凸显，国际投资和贸易低迷，保护主义、内顾倾向抬头。国内经济稳定运行的基础还不牢固，仍面临不少突出矛盾和问题。一是实体经济结构性供需失衡。供给体系产能大多数只能满足中低端、低质量、低价格的需求，随着消费结构加快升级，出口需求和投资需求相对下降，供给结构不适应需求新变化。二是有效需求增长乏力。有效投资特别是民间投资、制造业投资增速较低。经济下行对就业、收入、消费的滞后影响开始显现。国际需求持续低迷，国内综合要素成本不断上涨，产业和订单向外转移加快，我国外贸发展面临的形势更加复杂严峻。三是部分区域、行业、企业困难较大。一些资源型和传统产业比重大的地区，下行压力和发展困难较大。部分行业分化调整的影响，可能沿产业链传导。受市场环境偏紧和经营成本较高双重挤压，企业盈利能力较弱、亏损面较大。四是部分领域风险仍在积累。一些地区财政收支矛盾突出，防控金融风险力度需进一步加大，房地产市场仍面临结构性高房价、高库存双重挑战。五是大气雾霾等环境问题仍然突出。多频次、广区域、长时间、重浓度的雾霾天气，给人民生产生活带来较大影响。与此同时，安全生产、食品药品质量和民生等领域也出现一些新问题。我们要高度重视，准确分析研判国内外形势，增强忧患意识，强化底线思维，保持战略定力，更加有效应对。

　　**二、**2017年经济社会发展的总体要求、主要目标和政策取向
　　2017年是实施“十三五”规划的重要一年，是供给侧结构性改革的深化之年。中国共产党第十九次全国代表大会将要召开，具有重大而深远意义。做好2017年经济工作至关重要。
　　（一）总体要求
　　在以习近平同志为核心的党中央领导下，高举中国特色社会主义伟大旗帜，全面贯彻党的十八大和十八届三中、四中、五中、六中全会精神，以邓小平理论、“三个代表”重要思想、科学发展观为指导，深入贯彻习近平总书记系列重要讲话精神和治国理政新理念新思想新战略，统筹推进“五位一体”总体布局和协调推进“四个全面”战略布局，坚持稳中求进工作总基调，牢固树立和贯彻落实新发展理念，适应把握引领经济发展新常态，坚持以提高发展质量和效益为中心，坚持宏观政策要稳、产业政策要准、微观政策要活、改革政策要实、社会政策要托底的政策思路，坚持以推进供给侧结构性改革为主线，适度扩大总需求，加强预期引导，深化创新驱动，全面做好稳增长、促改革、调结构、惠民生、防风险各项工作，保持经济平稳健康发展和社会和谐稳定，以优异成绩迎接党的十九大胜利召开。
　　（二）主要预期目标
　　按照上述总体要求，综合考虑需要和可能，提出2017年经济社会发展的主要预期目标：
　　--经济增速保持在合理区间。国内生产总值预期增长6.5%左右，在实际工作中争取更好结果。主要考虑：一是符合客观实际。我国经济发展已经进入新常态，经济增速从高速增长转向中高速增长。2016年我国经济总量超过74万亿元，今年预期6.5%左右的增量，超过2016年6.7%的增量，同世界主要国家相比也是很高的速度。二是符合全面建成小康社会目标要求。与“十三五”规划目标相衔接，在2016年已经实现6.7%增长的基础上，今后4年，只要年均增长6.5%左右，就可以实现2020年国内生产总值比2010年翻一番的目标。三是能够满足就业目标需要。保持经济发展速度、推动经济发展，根本还是要不断解决好人民群众普遍关心的突出问题。四是有利于形成良好社会预期。2017年面临的国内外环境仍然复杂多变，要引导各方面更加注重坚持以提高发展质量和效益为中心，集中精力推动结构优化升级，积极应对各种不确定因素影响，为深入推进供给侧结构性改革创造良好环境。

　　--就业形势保持总体稳定。城镇新增就业1100万人以上，比2016年预期目标多100万人，城镇登记失业率控制在4.5%以内。主要考虑：一是就业压力较大。预计2017年仍有1500万左右新成长劳动力进入市场，加上去产能行业职工转岗需求有所增加，考虑退休等腾退的城镇就业岗位，需要新增约1100万人的城镇就业。二是就业容量不断扩大。随着产业结构调整，特别是创新创业和服务业发展，就业弹性系数明显提高，6.5%左右的经济增长，可以实现就业目标，实际完成数量还可以力争更多一些。三是实现城乡居民收入增长与经济增长基本同步的需要。体现了党和政府以人民为中心的发展思想和民生为先、就业为本的执政理念，有利于稳定预期和增强发展信心。
　　--价格总水平基本稳定。居民消费价格涨幅预期目标为3%左右。主要考虑：一是2017年翘尾因素约为0.6%，与去年基本持平。二是2016年9月以来，工业生产者出厂价格指数（PPI）由负转正，工业领域通缩压力有所缓解，但PPI向下游产业和终端消费传导，以及进口商品价格传导和国际大宗商品价格上升走势的影响，将会形成新的涨价因素。三是为价格改革预留出空间。
　　--国际收支保持基本平衡。继续促进货物贸易回稳向好，服务贸易较快增长，利用外资规模保持稳定，对外投资健康发展。主要考虑：一是世界经济和贸易增长低迷的状况短期内难以改变，但随着促进外贸稳增长调结构政策成效进一步显现，货物进出口有望继续回稳向好。二是电信、计算机和信息等高附加值服务出口继续较快增长，服务贸易结构将更加优化。三是大力营造公平、透明、可预期的法治化市场营商环境，利用外资将保持稳定，质量将进一步提高。四是对外投资活跃，为避免非理性投资引发经营、债务等风险，应稳妥有序推进对外投资。
　　--发展的质量和效益进一步提高。消费对经济增长的贡献率提高。农业基础进一步加强，去产能工作扎实推进，战略性新兴产业加快发展，服务业增加值占国内生产总值的比重继续提高。研究与试验发展经费投入强度和科技进步贡献率进一步提高。单位国内生产总值能耗下降3.4%以上、二氧化碳排放下降4.0%，主要污染物排放量继续下降，大气、水环境质量进一步改善。主要考虑：贯彻创新、协调、绿色、开放、共享的发展理念，促进产业转型升级、提质增效，全面提高产品和服务质量，加强节能环保和生态建设，符合推进供给侧结构性改革的要求，有利于创造新的增长点，提高经济增长潜力，推动经济保持中高速增长、迈向中高端水平。

　　--民生福祉进一步改善。农村贫困人口再减少1000万人以上，完成340万人易地扶贫搬迁建设任务。户籍人口城镇化率提高1个百分点以上，农业转移人口和其他常住人口在城镇落户1300万人以上。教育卫生文化等基本公共服务均等化水平进一步提高，义务教育巩固率和高中阶段教育毛入学率稳步提高。社会保障制度进一步完善，覆盖面继续扩大，保障水平适度提升。城乡居民基本医保人均财政补助标准提高到450元，大病保险筹资水平进一步提高。人口自然增长率7.5‰左右。主要考虑：全面落实以人民为中心的发展思想，在学有所教、劳有所得、病有所医、老有所养、住有所居上持续取得新进展。

　　（三）宏观经济政策取向
　　中央经济工作会议强调，稳中求进工作总基调是治国理政的重要原则，也是做好经济工作的方法论。2017年贯彻好这个总基调具有特别重要的意义。稳是主基调，稳是大局，在稳的前提下要在关键领域有所进取，在把握好度的前提下奋发有为。实现2017年经济社会发展目标，要坚持稳中求进工作总基调，保持宏观经济政策连续性和稳定性，继续实施积极的财政政策和稳健的货币政策。财政政策要更加积极有效，今年赤字率拟按3%安排，财政赤字2.38万亿元，比去年增加2000亿元。货币政策要保持稳健中性，今年广义货币M2和社会融资规模余额预期增长均为12%左右。
　　同时，加强各方面政策协调配合，统筹协调运用好中国特色宏观调控政策，更好发挥财政、货币、改革、产业、创新、就业、区域、投资、消费、贸易、价格、土地、环保等政策的协同组合效应，提高政策的精准性和有效性。
　　以财政、货币政策为支撑，投资、消费、贸易等政策为配合，供给侧结构性改革政策发力，着力化解供需结构性矛盾。大力实施减税降费政策，充分发挥中央财政资金“四两拨千斤”的引导带动作用，持续推进“放管服”改革、国企改革、价格改革，强化精准产业政策指导，着力纾缓实体经济困难。加大财政金融政策支持，实施差别化区域政策，注重就业和社会保障政策兜底，着力促进区域协调协同发展。深入推进农业供给侧结构性改革，完善农业补贴、投资、信贷、保险、用地等政策，支持农业技术创新，着力解决三农领域问题。通过稳健的货币政策、精准的产业政策和土地政策，深化金融体制改革，强化市场监管，防范和化解财政金融风险，着力防控资金脱实向虚和房地产波动风险。加大政策解读和信息发布力度，进一步完善产权保护制度，着力引导形成良好社会预期。具体工作中，注重聚焦“6个关键领域”着力攻坚，带动全局发展。
　　一是聚焦制度创新激发市场活力。深化重要领域和关键环节改革，强化体制机制创新，增加制度供给，有针对性解决制约经济社会发展的突出矛盾和问题，提高改革整体效能，力求取得新的突破。
　　二是聚焦振兴实体经济提升供给水平。深入推进“三去一降一补”，加快产业政策创新转型，优化生产力布局，努力实现供给体系由中低端产品为主向适应需求变化的中高端产品为主转变。
　　三是聚焦创新驱动培育壮大新动能。全面实施创新驱动发展战略，完善创新支持政策体系，着力增加创新资源供给，深入推进大众创业万众创新，促进发展动能接续转换，提高全要素生产率。
　　四是聚焦扩大有效需求稳定宏观环境。顺应需求升级大趋势，努力增加高品质产品和服务供给，提高政府投资效率，激发民间投资、社会资本活力，促进供给结构和需求结构有效匹配、消费升级和有效投资良性互动，实现更高质量、更有效率、更加公平、更可持续的发展。
　　五是聚焦重点领域重点区域有效防范化解风险。增强忧患意识，坚持底线思维，以防范金融风险、企业杠杆率过高、房地产市场泡沫积累、地方政府性债务及基层财政困难等风险为重点，创新完善风险处置方式，坚决守住底线，维护社会大局稳定。
　　六是聚焦民生改善促进社会和谐。落实以人民为中心的发展思想，着力化解结构性就业矛盾，推进精准扶贫精准脱贫，提高基本公共服务水平，加快发展教育、养老、卫生计生、文化、体育等社会事业，采取有力有效措施，改善生态环境，提升人民群众获得感和幸福度。

　　**三、**2017年经济社会发展的主要任务
　　2017年，将坚持稳中求进工作总基调，紧紧围绕供给侧结构性改革这条主线，贯彻新发展理念，引领经济新常态，着力抓好9方面工作。
　　（一）推动“三去一降一补”重点任务取得实质性进展。贯彻落实党中央、国务院关于供给侧结构性改革的总体部署，深入推进五大重点任务，减少无效低效供给、扩大有效供给，提高盈利能力。
　　一是扎扎实实化解过剩产能。今年再压减钢铁产能5000万吨左右，退出煤炭产能1.5亿吨以上。同时，淘汰、停建、缓建煤电产能5000万千瓦以上，以防范化解煤电产能过剩风险，提高煤电行业效率，优化能源结构，为清洁能源发展腾空间。充分运用市场化法治化手段，统筹处理好去产能与稳定供应、优化结构、转型升级的关系。将处置“僵尸企业”作为化解过剩产能的“牛鼻子”，严格执行环保、能耗、质量、安全等法律法规和标准，更加严格控制新增产能，更加坚决淘汰落后产能，更加严厉打击违法违规行为。创造条件推动企业兼并重组，妥善处置企业债务，做好人员安置工作。对符合条件的职工分流安置继续给予财政奖补资金支持。
　　二是坚持因城因地施策去库存。因地制宜、多种方式提高棚改货币化安置比例，加快实施1亿非户籍人口在城市落户方案，支持城镇居民改善性住房需求，规范发展住房租赁市场。按照“房子是用来住的、不是用来炒的”的定位，分类调控、因城施策，综合运用金融、土地、财税、投资、立法等手段，加快建立和完善促进房地产市场平稳健康发展长效机制，遏制热点城市房价过快上涨。研究按照建立住宅政策性金融机构的方向，探讨住房公积金制度改革，支持居民自住需求。
　　三是积极稳妥降低企业杠杆率。通过兼并重组、依法破产等途径，推动“僵尸企业”出清。支持银行、实施机构和企业通过自主协商开展市场化债转股。发展股权融资，加强疏通股本融资渠道，强化企业自身财务杠杆约束。
　　四是持续综合施策降低实体经济成本。进一步减少审批环节，清理取消行政审批中介服务违规收费，降低不合理中介评估费用，降低企业制度性交易成本。在减税、降费、降低要素成本上加大工作力度，大幅降低非税负担，降低融资成本，降低企业用能用地成本，降低物流成本，提高劳动力市场灵活性，支持企业内部挖潜、降本增效。深入开展涉企收费清理规范工作，落实好减税降费等各项惠企政策，减少政府定价的涉企经营性收费，进一步清理规范政府性基金、行政事业性收费和经营服务性收费，推动降低金融、铁路货运等领域涉企经营性收费，创新涉企收费监管制度，加强对市场调节类经营性收费的监管。继续加大工程建设领域保证金清理力度。
　　五是提高补短板的精准性和有效性。适当增加中央预算内投资，调整优化投资结构，集中用于脱贫攻坚、农业、灾后水利薄弱环节、软硬基础设施、创新能力建设等补短板重点领域。全面推进“十三五”规划《纲要》明确的165项重大工程项目。
　　（二）深入推进农业供给侧结构性改革。优化农业产业体系、生产体系和经营体系，加快培育农业农村发展新动能，狠抓农产品标准化生产、品牌创建、质量安全监管，促进农业提质增效、可持续发展。2017年粮食总产量稳定在5.5亿吨以上。
　　一是调好调顺调优农业结构。支持粮食、棉花、油料、糖料、天然橡胶等重要农产品生产基地建设和畜牧水产标准化规模养殖，加快国家级育制种基地和种养业良种工程建设。全面落实永久基本农田特殊保护政策，划定并建设粮食生产功能区、重要农产品生产保护区，制定特色农产品优势区建设规划，继续开展粮改饲、粮改豆试点，继续调减非优势产区籽粒玉米，扩大青贮玉米、苜蓿等优质牧草生产。稳定生猪生产并推动布局优化，提高乳制品质量。推进水产养殖减量增效，养护长江等重点水域水生生物资源。大力推进农产品标准化生产、品牌创建和保护，实施农产品质量安全工程。推进耕地轮作休耕制度试点，促进耕地草原河湖休养生息。深入实施化肥农药使用量零增长行动，推进畜禽养殖废弃物无害化处理和资源化利用。开展农业循环经济试点和工农复合型循环经济示范区（基地）建设，推进农业可持续发展试验示范区创建。
　　二是持续加强农业农村基础设施建设。继续推进172项重大水利工程建设，力争再新开工15项。加快高标准农田、农田水利、农村饮水安全巩固提升、现代种业、规模化养殖场、粮油仓储物流设施、公益性农产品批发市场等建设。启动农业、林业领域政府和社会资本合作（PPP）试点。实施农村新能源行动，支持中西部农村等基础薄弱区域信息基础设施和高效节水灌溉设施建设，加快完善县乡村物流网络。
　　三是加快农业科技创新。培育改良优质专用营养农畜品种，大力开发推广绿色高效种养技术，支持食品等加工业技术改造。建设一批农业重点实验室和国家农业科学实验站。完善农业科技创新激励机制，深入推进科研成果权益改革试点。推进基层农技推广体系改革，探索公益性机构和农技人员参与经营性服务的新机制，推动节本降耗、效益强农。启动智慧农业引领工程，全面实施信息进村入户工程，扩大农业物联网试验示范范围。
　　四是发展壮大农村新产业新业态。深入推进农村产业融合发展试点示范工程，引导二三产业向县城、重点乡镇及产业园区集中，启动建设“生产+加工+科技”的现代农业产业园。支持符合条件的涉农企业发行企业债券，建设产业融合发展项目。落实“互联网+”现代农业三年行动方案。深入开展电子商务进农村综合示范。推进农业与旅游、文化、康养等产业深度融合，建设一批产业文化旅游“三位一体”、生产生活生态同步改善、一二三产业深度融合的特色村镇。
　　五是加快深化农业农村改革。积极稳妥推进粮食等重要农产品价格形成机制和收储制度改革。坚持并完善稻谷、小麦最低收购价政策，合理调整最低收购价水平，形成合理比价关系。健全东北地区玉米市场化收购加补贴机制，稳步有序推进政策性粮食库存消化。完善棉花目标价格政策，调整大豆目标价格政策。完善农业补贴制度，落实好以绿色生态为导向的农业补贴制度改革。全面推进农业水价综合改革。深化农村集体产权制度改革，明晰农村集体产权归属，赋予农民更加充分的财产权利。改革财政支农投入机制，推动涉农资金整合。贯彻落实承包土地“三权分置”办法，统筹推进农村土地征收、集体经营性用地入市、宅基地制度改革试点。培育壮大农业新型经营主体和服务主体，发展多种形式的土地适度规模经营。在部分地区对适度规模经营农户实施大灾保险。完善农业再保险体系，以持续稳健的农业保险助力现代农业发展。
　　（三）进一步释放国内需求潜力。注重创新供给激活需求，夯实内需基础。
　　一方面，促进居民消费扩大和升级。持续推进“十大扩消费行动”，顺应消费需求变化新趋势，以改革创新增加消费领域特别是服务消费和绿色消费有效供给，保持消费平稳增长。2017年社会消费品零售总额预期增长10%左右。放宽服务业准入限制和社会领域投资管理。积极发展医养结合、文化创意、全域旅游等新兴消费，支持社会力量提供教育、文化、养老、医疗等服务。促进数字家庭、在线教育、虚拟现实等信息消费。开展加快内贸流通创新推动供给侧结构性改革扩大消费专项行动，推动实体店销售和网购融合发展。保护和传承中华老字号。扩大内外销产品“同线同标同质”实施范围。大力发展冷链物流。建立健全食品药品等的质量安全标准和追溯体系。加大价格监管和反垄断执法力度，保护消费者合法权益。强化收入分配改革激励导向，实施重点群体激励计划，开展城乡居民增收综合配套政策试点。

　　另一方面，精准扩大有效投资。加大关键领域和薄弱环节补短板工作力度，带动有效投资的扩大，增加有效供给。2017年全社会固定资产投资预期增长9.0%左右。拟安排中央预算内投资5076亿元，集中用于重大战略、重大工程和重点建设任务。着力激发民间投资活力，认真落实有关鼓励支持政策，向民间资本进一步放开基础电信领域竞争性业务、油气勘探开发、国防科技等领域的市场准入。创新投融资方式，继续规范推广政府和社会资本合作，筛选和推介一批有较好现金流、有稳定回报预期的项目。强化投资监管，创新稽察监管方式，实行网上全面监测，深化现场稽察。
　　（四）持续深化重要领域和关键环节改革。抓好已出台改革举措落地实施，更好发挥改革牵引作用。
　　一是全力推进简政放权放管结合优化服务改革。再削减一批行政审批事项，全面清理调整生产许可证、经营许可证。推动关联、相近类别审批事项整体取消或下放。制定工商登记前置审批事项清单和企业设立后的经营许可清单，确需保留的实行多证合一，扩大“证照分离”改革试点。实现“双随机、一公开”监管全覆盖，推进综合执法改革。大力推行“互联网+政务服务”，开展“减证便民”专项行动。按照登记机构、登记簿册、登记依据和信息平台“四统一”要求，进一步扩大不动产统一确权登记的覆盖面，基本建成覆盖全国的信息平台。
　　二是深化国企国资和重点行业改革。基本完成公司制改革。形成有效制衡的公司法人治理结构、灵活高效的市场化经营机制。加快国有资本投资、运营公司试点。推动电力、石油、天然气、铁路、民航、电信、军工等领域混合所有制改革试点。支持民营企业参与国有企业改革。抓好电力和石油天然气体制改革，有序放开发用电计划，扩大市场化交易规模。适度有序放开油气勘查开采等竞争性业务，完善油气进出口管理体制。完善食盐储备和监管机制。
　　三是深化投融资体制改革。贯彻落实[深化投融资体制改革的意见](https://www.pkulaw.com/chl/3ec7dc0f185a661bbdfb.html)、[企业投资项目核准和备案管理条例](https://www.pkulaw.com/chl/9545cd6cfc040dd1bdfb.html)、政府核准的投资项目目录（2016年本）。出台政府投资条例，出台[企业投资项目核准和备案管理办法](https://www.pkulaw.com/chl/a4b81e2a780f004abdfb.html)。支持在一定领域、区域内试点企业投资项目承诺制。开展金融机构持有企业股权试点，完善保险资金等机构资金对项目建设的投资机制。
　　四是纵深推进价格改革。在全国范围内基本完成省级电网输配电价改革，公布所有省级电网输配电价，启动东北、西北等区域电网输配电价改革试点，研究制定地方电网和新增配电网配电价格，推进具备条件的发电、售电价格通过市场形成。完善新能源价格机制和补贴办法。制定跨省天然气管道运输价格。继续推进医疗服务价格改革，全面推开城市公立医院医药价格改革，开展按病种收费工作。
　　五是营造公平竞争市场环境。落实[完善产权保护制度依法保护产权的意见](https://www.pkulaw.com/chl/0d3cd162234f304dbdfb.html)，加强产权保护制度建设。激发非公有制经济活力，积极构建新型政商关系。出台实施激发和保护企业家精神的意见。全面实施公平竞争审查制度，坚决遏制出台新的排除限制竞争的政策措施。严厉查处重大典型垄断案件，预防和制止垄断行为。推进社会信用体系建设。建立全国一体化的信用信息共享体系，扩大守信联合激励和失信联合惩戒领域。

　　六是稳妥推进财税和金融体制改革。加快推进中央与地方财政事权和支出责任划分改革。制定中央与地方收入划分总体方案。健全地方税体系。规范地方政府举债行为。稳妥推进金融监管体制改革，加强金融宏观审慎管理制度建设。进一步完善人民币汇率市场化形成机制。健全国有商业银行治理结构，深化多层次资本市场体系改革，推动《[证券法](https://www.pkulaw.com/chl/e3a5d596aa075797bdfb.html)》修订，完善股票发行制度改革。加快发展现代保险服务业。
　　（五）着力振兴实体经济。坚持创新驱动发展，以提高质量和核心竞争力为中心，坚持品牌引领升级，全面提高创新供给能力，促进新动能更快发展、新产业更快成长、传统产业更快改造提升、新旧动能加快接续转换。
　　一是加快培育壮大经济发展新动能。推动8个区域全面创新改革试验取得决定性进展，出台培育壮大新动能、互联网市场准入等政策文件。支持和引导分享经济发展，提高社会资源利用效率。建立国家科技决策咨询制度，改革科技评价制度，统筹推进中央财政科技计划和资金管理改革，开展以知识价值为导向的分配政策试点。加大对信息、民用空间、重大科技基础设施建设投入，支持北京怀柔、上海张江、安徽合肥综合性国家科学中心建设，建成一批产业创新中心，构建全国一体化的国家大数据中心体系。继续布局建设国家自主创新示范区。深入推进协同创新。扩大区域性成果转移转化试点示范，创建国家可持续发展议程创新示范区。继续实施国家科技重大专项，启动实施科技创新2030-重大项目。在重大创新领域启动组建国家实验室，建设一批国家技术创新中心。支持北京、上海建设科技创新中心。新建一批双创示范基地和专业化众创空间。实施院所、企业和社团三大创新创业活动，围绕创新创业平台市场化运行、创业人才培养等瓶颈制约，出台一批硬措施。发挥好创业投资引导基金作用，促进创业投资持续健康发展。打造全国双创活动周和“创响中国”品牌，持续营造良好氛围。
　　二是推动新兴产业持续健康发展。壮大战略性新兴产业，设立国家战略性产业发展基金，在新材料、新能源、人工智能、集成电路、生物制药、第五代移动通信等前沿战略性领域，发挥企业主体作用，以市场化方式布局一批系统性工程。推进“互联网+”和大数据重大工程，促进互联网、物联网、云计算与经济社会融合发展。进一步加大网络提速降费力度，降低创业创新成本促进产业升级。加强军民融合创新发展，推动太空、海洋和网络空间等领域军民融合重大示范项目建设。
　　三是促进传统产业加快改造提升。深入实施《中国制造2025》。落实装备制造业质量品牌提升专项行动、消费品工业“三品”专项行动、服务型制造专项行动和制造业与互联网融合发展专项行动。提高质量标准，广泛开展质量提升行动，加强全面质量管理，夯实质量技术基础，强化质量监督。弘扬工匠精神，厚植工匠文化，完善激励机制，培育“中国工匠”，打造“中国品牌”。推进国家智能制造示范区、制造业创新中心建设，继续实施绿色制造工程、工业强基工程和高端装备创新工程。夯实工业基础，持续实施制造业升级改造重大工程包，重点支持企业智能化改造、基础能力提升、绿色制造推广、高端装备发展等10大工程。推动汽车产业转型升级。实施国家新型工业化产业示范基地卓越提升计划。推动原材料、交通、能源等基础产业转型升级，加快建设现代综合交通运输体系，全面实施能源生产和消费革命战略。
　　四是推进服务经济创新发展。研究出台服务经济创新发展大纲，深入实施新一轮服务业综合改革试点，加快培育服务业新业态新模式。开展服务业补短板专项行动，提升服务质量，推进标准化发展，强化知识产权服务。推进高技术服务业创新工程。
　　（六）以三大战略引领区域协调协同发展。充分发挥各地区比较优势，实施差别化区域发展政策，加快培育形成新的增长轴、增长带。
　　一是扎实推进三大战略。贯彻落实习近平总书记在推进“一带一路”建设工作座谈会上的重要讲话精神，聚焦重点地区、重点国家、重大标志性项目，积极开展务实合作，推动合作协议和合作规划实施。疏控结合推进北京非首都功能有序疏解，协调加快北京城市副中心等功能承接平台规划建设，持续推进交通、生态、产业三个重点领域率先突破，强化创新、改革、试点三个关键支撑，推进基本公共服务共建共享，支持北京服务业深化改革扩大开放综合试点。坚持生态优先、绿色发展，实施长江经济带建设水环境治理、水生态修复、水资源保护工程，推动航道畅通、枢纽互通、江海联通、关检直通，推进黄金水道重大项目，建立负面清单管理制度。
　　二是优化区域发展格局。全面实施西部大开发“十三五”规划，加强基础设施薄弱环节建设和生态环境保护，加快特色优势产业转型升级发展。全面实施新一轮东北地区等老工业基地振兴重大政策和重点任务，推动实施东北振兴“十三五”规划，组织东北地区和东部地区部分省市开展对口合作，加大对城区老工业区、独立工矿区改造和采煤沉陷区综合治理支持力度。全面实施促进中部地区崛起“十三五”规划，支持汉江、淮河生态经济带加快发展，办好山西可持续发展综改试验区。支持东部地区加快率先转型发展，进一步发挥改革创新示范引领作用。统筹加强国内国际区域合作，支持重要功能平台建设。大力推进陆海统筹发展。加快推进革命老区、民族地区、边疆地区和集中连片特困地区基础设施和公共服务设施建设，推动融入国家重大发展战略。加大中央支持和对口支援力度，继续支持新疆、西藏和四省藏区发展。

　　三是扎实推进新型城镇化。以人的城镇化为核心，深化户籍制度改革，推动居住证制度全覆盖，将农业转移人口有序纳入住房公积金缴存覆盖范围，提高城镇化质量。加快编制粤港澳大湾区、海峡西岸、关中平原、呼包鄂榆等城市群发展规划，启动国家中心城市布局建设，推动一批具备条件的县和特大镇有序设市。促进“多规合一”，提升城市规划设计水平。加快城市市政基础设施改造与建设，加强城市地质调查，稳步推进地下综合管廊建设和地下管线综合管理，有效治理交通拥堵等“城市病”。深入推进美丽特色小（城）镇、美丽乡村建设。发挥新型城镇化对新农村建设、农村一二三产业融合发展带动作用，推动易地扶贫搬迁与新型城镇化有机结合。

　　（七）推进更深层次更高水平的双向开放。加强统筹谋划，注重协调内外，把扩大开放、优化服务、强化监管有效结合起来，培育国际竞争合作新优势。
　　一是更加扎实有效推进“一带一路”建设。加快推进六大国际经济合作走廊建设，实施好瓜达尔、比雷埃夫斯、汉班托塔等重要港口建设运营，稳步推进印尼雅万高铁、俄罗斯莫喀高铁、中老铁路、中泰铁路、马新铁路、匈塞铁路等项目，进一步打造中欧班列统一品牌。高质量办好“一带一路”国际合作高峰论坛。积极务实推进国际产能合作，完善产能合作重点项目库，推进产能合作示范区建设，带动装备、技术、标准、服务走出去。重点推动中白工业园、埃及苏伊士经贸合作区、马中关丹产业园等境外经贸产业园区建设。继续推进跨境（边境）经济合作区建设。推动网上丝绸之路建设合作。加强教育、科技、文化、卫生、旅游等人文交流合作。
　　二是推动对外贸易继续回稳向好。落实和完善促进外贸发展政策，充分发挥出口信用保险作用，对成套设备出口融资应保尽保。设立服务贸易创新发展引导基金。继续开展市场采购贸易、外贸综合服务企业等试点，推进优势农产品走出去。加快外贸转型升级示范基地建设。支持加工贸易向产业链高端延伸、向中西部地区梯度转移。提升贸易便利化水平，在全国范围内推广国际贸易单一窗口建设，实现全国通关一体化。增加先进技术设备和关键零部件进口。推进区域全面经济伙伴关系协定谈判，继续推进亚太自贸区建设。妥善应对贸易摩擦，维护我国合法权益。
　　三是进一步优化外资营商环境。出台实施扩大开放积极利用外资若干措施、新版[外商投资产业指导目录](https://www.pkulaw.com/chl/4804e2412f5e1853bdfb.html)、[中西部地区外商投资优势产业目录](https://www.pkulaw.com/chl/c2eb5f655fba5647bdfb.html)，进一步扩大开放领域，简化管理程序。更加积极推进自由贸易试验区建设，围绕重点任务和薄弱环节继续深化改革探索，力争取得更多可复制推广的制度创新成果。完善外商投资企业外债管理制度。2017年非金融类外商直接投资预期与2016年持平。

　　四是引导对外投资健康有序发展。加强对外投资活动的真实性审查，建立不良记录名单制度，实施国有企业对外投资资本金制度，防范对外投资过快增长风险。健全对外投资促进政策和服务体系，引导企业有序合规走出去。2017年非金融类境外直接投资预期与2016年基本持平。
　　（八）加快推动绿色发展。深入推进生态文明建设，全面促进资源节约，扩大生态产品供给，推动环境质量总体改善，加快建设美丽中国。
　　一是深化生态文明体制改革。积极推进国家生态文明试验区建设。开展健全国家自然资源资产管理体制试点。全面推进划定并严守生态保护红线，推进实施全民所有自然资源资产有偿使用制度改革，制定领导干部自然资源资产离任审计暂行规定。开展生态文明建设目标评价，发布2016年度省级地区绿色发展指数。实现中央环境保护督察全覆盖，推进省以下环保机构监测监察执法垂直管理制度改革试点。推动生态环境损害赔偿制度改革。健全绿色金融体系，加大绿色债券发行力度。
　　二是完善主体功能区制度。制定完善主体功能区战略和制度的意见。启动全国和各省（区、市）主体功能区规划修订工作。加快推动省级空间规划试点，制定编制市县空间规划指导意见。制定落实生态保护红线、环境质量底线、资源利用上线和环境准入负面清单的技术规范，推动战略和规划环评落地。制定建立国家公园体制总体方案。制定建立资源环境承载能力监测预警长效机制的意见，在长江经济带开展监测预警。严格实行重点生态功能区产业准入负面清单制度。制定海洋主体功能区建设配套政策。开展省级国土规划编制。
　　三是进一步提高资源利用效率。落实“十三五”节能减排综合工作方案。开展能源、水、土地资源消耗总量和强度“双控”，实施全民节能行动、重点用能单位“百千万”行动和能效“领跑者”引领行动，推进用能权有偿使用和交易试点，实施多能互补系统集成优化工程，减少弃水弃风弃光。推进全民节水行动，建立水效标识制度，加大非常规水源利用力度。实施循环发展引领行动，制定“互联网+”资源循环行动方案，支持园区循环化改造、资源循环利用产业示范基地、废弃物综合利用基地建设。
　　四是强化环境治理和生态保护。深入实施大气、水、土壤污染治理“三大行动计划”。大力推进大气污染治理，完善重污染天气预警机制，实施京津冀地区大气污染防治强化措施，开展京津冀及周边、长三角、珠三角等重点地区和重点城市煤炭消费减量替代和污染防治，推进北方地区冬季清洁取暖，全面实施散煤综合治理，完成以电代煤、以气代煤300万户以上。建立水污染防治预警和综合督导机制，制定实施重点流域水污染防治“十三五”规划、长江经济带生态环境保护规划。创新河湖管护体制机制，全面推行河长制。开展城市生态修复试点示范，全面推进城市生态修复。加大城市黑臭水体治理力度，提升城镇污水处理再生利用设施系统整体效能。推进重点城市实施生活垃圾强制分类制度，加快建设垃圾分类投放、收运和处理设施。稳步推进控制污染物排放许可制，开展农村生活垃圾分类和资源化利用百县示范。建立以排污许可证管理为核心的固定污染源环境管理制度体系，严控入河排污总量。积极开展环境污染第三方治理。全面开展全国土壤污染状况详查，加快推动土壤污染防治法立法，制定或修订农用地、建设用地土壤环境质量等标准。实施工业污染源全面达标排放计划。全面开展国家生态安全屏障建设，积极推进大规模国土绿化行动，实施山水林田湖生态保护和修复工程、生物多样性保护重大工程。继续实施退耕还林还草、退牧还草、重点防护林体系、天然林资源保护、水土流失综合治理等工程，开展流域水环境综合治理与可持续发展试点示范，推进退耕还湿、退养还滩。严格管控围填海。建立自然保护区遥感监测体系，强化自然保护区监管。
　　五是积极应对气候变化。启动全国碳排放权交易市场。加快推进低碳试点示范、气候适应型城市建设试点。积极参与和引导《巴黎协定》后续谈判，精心运筹气候外交，做好国内履约工作。继续推动国际对话和务实合作，加强气候变化南南合作。
　　（九）更加注重保障和改善民生。更好统筹民生改善与经济发展，织密扎牢民生保障网，努力提高公共服务水平和质量，持续增强人民群众获得感。
　　一是全力推进脱贫攻坚。巩固深化脱贫攻坚成果，加快实施易地扶贫搬迁、产业扶贫、转移就业扶贫等脱贫攻坚重点工程，大力实施以工代赈，支持贫困地区交通、水利、教育、卫生计生、文化、社会保障等基础设施和基本公共服务建设。研究支持边境居民脱贫奔小康、稳边固边政策措施。支持川陕等革命老区、赣南和闽粤原中央苏区开发建设与脱贫攻坚。稳妥推进贫困地区水电矿产资源开发资产收益扶贫改革试点。深入开展金融扶贫、健康扶贫、教育扶贫、交通扶贫、旅游扶贫、电商扶贫，做好因病等致贫返贫群众帮扶。推动网络扶贫试点省建设。
　　二是深入实施就业优先战略。持续推进重点群体就业创业。实施好高校毕业生就业促进、创业引领、基层成长等计划，切实做好退役军人安置工作。围绕培育返乡创业产业集群、发展农村电商、化解过剩产能发展接续产业等重点，继续扩大支持农民工等人员返乡创业试点覆盖范围。妥善做好煤炭、钢铁行业化解过剩产能过程中职工分流安置工作。加强基层就业服务设施和公共实训基地建设，强化就业援助，提高公共就业服务能力。
　　三是强化社保兜底。推动基本养老保险制度改革和完善工作。加快城乡居民医疗保险制度整合。开展长期护理保险试点、生育保险和职工基本医疗保险合并实施试点。统筹推进社会救助体系建设。健全购租并举的住房制度，持续改善中低收入群众和困难群众住房条件。2017年，棚户区住房改造新开工600万套。继续推进农村危房改造，重点支持低保户、农村分散供养特困人员、贫困残疾人家庭和建档立卡贫困户。继续推进公租房等保障房建设。
　　四是健全基本公共服务体系。实施“十三五”推进基本公共服务均等化规划，建立基本公共服务清单制。改革高等教育招生计划管理，2017年，普通高等教育本专科招生735万人，研究生招生84.4万人。推进世界一流大学和一流学科建设。全面推开公立医院综合改革，全面启动多种形式的医疗联合体建设试点，建立促进优质医疗资源上下贯通的考核和激励机制。落实政府办医责任，实施全民健康保障工程，提升中西部县域内医疗卫生综合服务能力，加强疾病防控、妇幼保健机构能力建设。适应实施全面两孩政策，加强生育医疗保健服务。依法支持中医药事业发展。出台实施“十三五”国家老龄事业发展和养老体系建设规划。健全养老服务体系，实施养老护理人员培训计划。出台实施国家人口发展规划，推进落实全面两孩配套政策。切实加强妇女、未成年人、残疾人等社会群体权益保护。提高基本公共文化服务均等化水平，推动地方落实国家基本公共文化服务指导标准。推动创新公共文化服务供给方式，鼓励社会力量参与公共文化服务体系建设。繁荣发展哲学社会科学。实施全民健身计划，推进公共体育普及，推动实施足球中长期发展规划和足球场地设施建设规划。
　　深化内地与香港、澳门的投资经贸合作，推动内地与港澳关于建立更紧密经贸关系安排（CEPA）升级。支持港澳参与和助力“一带一路”建设、区域经济合作，落实“十三五”规划《纲要》涉港澳工作。积极推进港珠澳大桥建设，促进粤港澳三地加快基础设施互联互通。加快前海、南沙、横琴等粤港澳合作平台建设，推动泛珠三角区域合作向更高层次、更深领域发展。支持香港发展创新和科技事业，巩固提升国际金融、航运、贸易三大中心地位，培育发展新优势。进一步支持澳门建设世界旅游休闲中心、中国与葡语国家商贸合作服务平台，支持澳门发展会展、特色金融、文化创意等产业和跨境电子商务，促进澳门经济适度多元发展。建设好平潭综合实验区、福州新区、中国（福建）自由贸易试验区等合作平台，深化海峡两岸经济交流合作。
　　各位代表，做好2017年经济社会发展各项工作，任务艰巨，意义重大。我们要更加紧密地团结在以习近平同志为核心的党中央周围，高举中国特色社会主义伟大旗帜，以邓小平理论、“三个代表”重要思想、科学发展观为指导，深入贯彻习近平总书记系列重要讲话精神和治国理政新理念新思想新战略，自觉接受全国人大监督，虚心听取全国政协意见建议，凝心聚力、改革创新，奋发有为、攻坚克难，促进经济社会平稳健康发展，以优异成绩迎接党的十九大胜利召开！

　　注1：经十二届全国人大四次会议审议通过的《2016年国民经济和社会发展计划总表》总计包括62个指标，其中：预期性指标43个，约束性指标19个；加☆的15个指标为《[中华人民共和国国民经济和社会发展第十三个五年规划纲要](https://www.pkulaw.com/chl/3fd7b48d6cb8d951bdfb.html)》确定的约束性指标。
　　注2：预期性指标的计划目标是期望的发展目标，是对指标的预期值，不是指令性的，也不是预测值，实际结果可能高一点，也可能低一点。约束性指标的计划目标具有指令性和刚性，体现国家的宏观调控意图，是必须完成的目标。
　　注3：约束性指标的完成情况用是否完成进行评价；预期性指标完成情况的评价以偏离计划值10%为标准，高于计划值110%的为“高于预期”，低于计划值90%的为“低于预期”，两者之间为“符合预期”；但指标带有限高或限低性质的除外，如城镇登记失业率指标2016年实际数为4.02%，低于计划值的90%，评价仍为“符合预期”。
　　注4：加#的指标数据仍为2016年预计值，待相关部门最终核实后再行调整为全年实际值；个别指标受2015年基数调整影响，2016年实际增速还可能有所变化。

第十二届全国人民代表大会财政经济委员会关于2016年国民经济和社会发展计划执行情况与2017年国民经济和社会发展计划草案的审查结果报告
　　（2017年3月12日第十二届全国人民代表大会第五次会议主席团第二次会议通过）

十二届全国人大五次会议主席团：
　　第十二届全国人民代表大会第五次会议审查了国务院提出的《关于2016年国民经济和社会发展计划执行情况与2017年国民经济和社会发展计划草案的报告》以及2017年国民经济和社会发展计划草案。全国人民代表大会财政经济委员会在对计划报告和计划草案初步审查的基础上，根据各代表团和有关专门委员会的审查意见，又作了进一步审查。国务院根据审查意见对计划报告作了修改。现将审查结果报告如下。
　　财政经济委员会认为，2016年国民经济和社会发展计划执行情况总体是好的。面对复杂严峻的国内外环境，在以习近平同志为核心的党中央坚强领导下，国务院和地方各级人民政府认真落实党中央决策部署和十二届全国人大四次会议提出的各项要求，坚持稳中求进工作总基调，深入贯彻落实新发展理念，主动把握和引领经济发展新常态，着力推进供给侧结构性改革，适度扩大总需求，妥善应对风险挑战，圆满完成全年主要目标任务，经济社会保持平稳健康发展，实现了“十三五”良好开局。经济运行稳中向好，经济结构继续优化，改革开放深入推进，发展新动能不断增强，就业增长超出预期，人民生活持续改善，生态环境有所好转，全面建成小康社会又取得新的重要进展，成绩来之不易。同时也要看到，经济社会发展仍面临不少困难和挑战。经济稳定运行基础还不牢固，供给和需求结构性矛盾突出；实体经济困难较多，经济增长内生动力不足；农业劳动生产率和比较收益较低，结构调整任务艰巨；金融风险隐患增加，企业杠杆率高企；房地产市场分化，热点城市高房价与三四线城市高库存并存；生态环境形势依然严峻，一些地区严重雾霾频发；在教育、医疗、养老、安全生产、食品药品安全、收入分配等方面仍存在不少矛盾和问题。对此，要高度重视，采取措施努力解决。
　　财政经济委员会认为，国务院提出的计划报告和2017年计划草案，符合中央经济工作会议精神和“十三五”规划纲要要求，符合我国经济社会发展实际，指导思想明确，重点突出，主要目标和工作安排总体可行，建议批准国务院提出的《关于2016年国民经济和社会发展计划执行情况与2017年国民经济和社会发展计划草案的报告》，批准2017年国民经济和社会发展计划草案。
　　财政经济委员会认为，2017年是实施“十三五”规划的重要一年，也是供给侧结构性改革的深化之年。今年将要召开具有重大而深远意义的中国共产党第十九次全国代表大会，做好经济社会工作至关重要。要在以习近平同志为核心的党中央领导下，高举中国特色社会主义伟大旗帜，全面贯彻党的十八大和十八届三中、四中、五中、六中全会以及中央经济工作会议精神，以邓小平理论、“三个代表”重要思想、科学发展观为指导，深入贯彻习近平总书记系列重要讲话精神和治国理政新理念新思想新战略，统筹推进“五位一体”总体布局和协调推进“四个全面”战略布局，坚持稳中求进工作总基调，牢固树立和贯彻落实新发展理念，适应把握引领经济发展新常态，坚持以提高发展质量和效益为中心，坚持宏观政策要稳、产业政策要准、微观政策要活、改革政策要实、社会政策要托底的政策思路，坚持以推进供给侧结构性改革为主线，适度扩大总需求，加强预期引导，深化创新驱动，全面做好稳增长、促改革、调结构、惠民生、防风险各项工作，保持经济平稳健康发展和社会和谐稳定，以优异成绩迎接党的十九大胜利召开。为此，财政经济委员会提出以下建议：
　　（一）着力振兴实体经济。贯彻落实供给侧结构性改革总体部署，进一步聚焦支持实体经济。坚持创新驱动发展，持续提升创新能力，加快培育壮大新兴产业，促进传统产业更快改造提升和新旧动能加快接续转换，不断提升供给结构对需求结构的适应性。推进制造业转型升级，促进制造业研发、生产、管理、服务等模式变革，全面提升制造业品牌建设、质量效益和服务水平。深入推进“三去一降一补”重点工作取得新进展。充分运用市场化法治化手段化解过剩产能，严格执行环保、土地、能耗、质量、安全、财务等法律法规和标准，抓住处置“僵尸企业”这个牛鼻子，推进企业兼并重组、债务重组或破产清算，着重做好下岗分流职工安置和不良资产处置。遏制非金融企业杠杆率上升势头，强化企业预算约束，降低资产负债率。加大减税降费特别是涉企收费清理力度，继续适当降低“五险一金”有关缴费比例，降低要素成本和各类交易成本特别是制度性交易成本，切实减轻企业负担。进一步释放国内需求潜力，适应消费升级需要，调整优化投资结构，提高补短板的精准性和有效性。
　　（二）加快培育农业农村发展新动能。深入推进农业供给侧结构性改革，积极调整农业结构，发展适度规模经营，着力提高农业综合效益和竞争力。严守耕地红线，扩大耕地轮作休耕制度试点规模，保护和提高粮食综合生产能力。深化农村集体产权制度改革，完善落实承包土地“三权分置”的政策措施，统筹推进农村土地征收、集体经营性建设用地入市、宅基地制度改革试点，赋予农民更加充分的财产权利。改革完善粮食等重要农产品价格形成机制和收储制度。加快完善农业补贴制度，提高补贴政策指向性和精准性。提高“三农”金融服务水平，完善农业保险体系。加大严格执法力度，切实抓好农产品质量安全。加强新型农民技能培训，多措并举拓展农民增收空间。强化脱贫攻坚支撑保障体系，健全脱贫攻坚责任制，帮助贫困群众解决实际问题，推动精准扶贫、精准脱贫各项政策措施落到实处。
　　（三）切实加强生态环境保护。深入实施大气、水、土壤污染防治行动计划，标本兼治，推动绿色低碳循环发展取得新进展。强力推进雾霾防治，持续削减污染物排放量，扎实推动北方地区冬季清洁取暖和建筑节能改造，使空气质量改善与人民群众切身感受相符。优化能源结构和利用方式，推动煤炭清洁高效利用，提高清洁能源消费比重，认真解决弃水弃风弃光问题。推进全国碳排放交易市场建设。加大污染排放高的重点行业治理力度，推行垃圾分类制度。加强环境执法能力建设，强化各级政府责任，依法严厉打击偷排偷放等各类环境违法违规行为。大力推进生态文明建设，加强生态保护修复，划定生态保护红线，完善生态补偿机制。加快发展节能环保产业，促进传统产业清洁生产、绿色改造，实现经济发展与环境保护双赢。
　　（四）更加注重保障和改善民生。坚持以人民为中心的发展思想，努力解决好人民群众普遍关心的突出问题。健全基本公共服务体系，推进基本公共服务均等化。调整优化教育结构，均衡配置教育资源，加大对与劳动力市场对接的职业教育和非学历教育培训的投入，统一城乡义务教育学生“两免一补”政策。发展文化事业，加强社会主义精神文明建设。深入实施就业优先战略，提高公共就业服务能力，持续推进重点群体就业创业。深化收入分配制度配套改革，加快推进综合与分类相结合的个人所得税改革，千方百计提高居民收入水平。健全养老服务体系，继续适当提高基本养老金标准，确保养老金按时足额发放，推动职工基础养老金全国统筹。完善基本医疗服务机制，实现异地就医住院费用直接结算。完善棚改安置和公租房分配方式，实行实物保障与货币补贴并举。科学合理制定城乡低保标准，落实社会救助标准与物价上涨挂钩联动机制，加强社会保险、救助和慈善等方面资源统筹，保障困难群众基本生活。进一步加大监管力度，切实保障食品药品安全。
　　（五）有效防控各种潜在风险。要保持稳健中性的货币政策，把防控金融风险放在更加重要的位置，防止货币供应过于宽松而产生的加杠杆效应和资产泡沫放大。要摸清风险隐患，着力抓好影子银行、互联网金融、地方债务、房地产等方面的风险防控，积极做好应对预案，妥善处置不良资产。更加重视对跨领域、跨市场风险的监管和监测，加快建立监管协调机制，抓好对资产管理市场的统一监管，有序打破刚性兑付，确保不发生系统性风险。坚持住房的居住属性，从实际出发，综合运用金融、土地、财税、投资等手段，依法加强住房市场监管和分类调控，控制信贷过度流向房地产业。深化土地制度改革，科学做好城市土地规划、供应，缓解部分城市供地紧张矛盾。加快研究建立符合国情、适应市场规律、促进房地产市场健康发展的长效机制和制度安排。
　　（六）积极推进关键性改革和高水平开放。全面落实十八大以来党中央部署的改革任务，坚持社会主义市场经济改革方向，统筹推进，重点突破，更好发挥改革牵引作用。加快转变政府职能，以清单管理推动简政放权，进一步减少行政干预，全面建立公平竞争审查制度，完善事中事后监管。深化国企国资改革，积极推进混合所有制改革，加快形成有效制衡的公司法人治理结构和灵活高效的市场化经营机制，在电力、石油、天然气、铁路、民航、电信、军工等领域取得实质性进展。遵循权利平等、机会平等、规则平等，进一步放宽市场准入，消除各种隐性壁垒，激发非公有制经济活力，稳定信心和预期，鼓励和促进民间投资。加强对各种所有制组织和自然人财产权的平等保护，着力营造法治、透明、可预期的体制政策环境，保护企业依法经营。发展多层次资本市场，扩大直接融资，增强金融服务实体经济的能力，努力缓解小微企业融资难融资贵。抓好重大改革顶层设计和统筹协调，充分调动地方和基层推动改革的积极性和主动性。加强涉及改革的法律立改废释工作，推动改革有序推进。深入推进“一带一路”建设和国际产能合作，加快推进高标准自贸试验区建设，打造更有国际竞争力的投资环境，防范对外投资过快增长风险，积极应对贸易保护主义挑战，促进国际贸易和投资自由化便利化，不断深化新一轮高水平对外开放。
　　以上报告，请予审议。

第十二届全国人民代表大会财政经济委员会
　　2017年3月12日

© 北大法宝：（[www.pkulaw.com](https://www.pkulaw.com/)) 专业提供法律信息、法学知识和法律软件领域各类解决方案。北大法宝为您提供丰富的参考资料，正式引用法规条文时请与标准文本核对。
欢迎查看所有[产品和服务](https://www.pkulaw.com/helps/2.html)。法宝快讯：[如何快速找到您需要的检索结果？](https://www.pkulaw.com/helps/70.html)[法宝](https://www.pkulaw.com/helps/69.html)V6有何新特色？

扫描二维码阅读原文

**原文链接：**<https://www.pkulaw.com/chl/2126997d44e26940bdfb.html>
